# Experience with recipient splenic artery inflow in adult liver transplantation: a case series

**DOI:** 10.1186/1757-1626-1-82

**Published:** 2008-08-11

**Authors:** Wesley B Vanderlan, Marwan S Abouljoud, Atsushi Yoshida, Dean Y Kim

**Affiliations:** 1 Department of Surgery, Division of Transplant Surgery and Hepatobiliary Surgery, Henry Ford Hospital, 2799 West Grand Boulevard (CFP-2), Detroit, MI, 48202, USA; 2 Department of Surgery, University of Michigan, 2207 Taubman Center, 1500 E Medical Center Drive, Ann Arbor, MI, 48109-0342, USA

## Abstract

**Introduction:**

Hepatic artery thrombosis following orthotopic liver transplant is one of the most common reasons for early graft failure. Meticulous reconstitution of hepatic artery flow remains essential for good outcomes. Prior surgery, body habitus, hepatic artery inadequacy and anatomic differences can complicate hepatic artery revascularization.

**Case presentation:**

We report a single institution's experience, from January 1996 to January 2007, using splenic artery inflow in seven patients with inadequate native hepatic arteries.

**Conclusion:**

End-to-side anastomosis was associated with postanastomotic intimal hyperplasia. End-to-end anastomosis provided effective hepatic inflow, demonstrated splenic and pancreatic safety, and was not associated with the intimal hyperplasia experienced with end-to-side anastomosis.

## Background

Hepatic artery thrombosis (HAT) is the most common vascular complication following orthotopic liver transplant (OLT) [1]. HAT often results in severe hepatocyte and bile duct necrosis leading to graft loss [1-3]. The causes of HAT are multifactorial and include intrinsic hepatic artery inadequacy, poor surgical technique, immunologic response and coagulopathies. Intrinsic hepatic artery inadequacy encompasses diminutive size, thrombosis, intimal disruption, aberrant anatomy, arteriosclerosis and retransplantation.

Hepatic artery reconstruction typically involves the creation of a conduit using the recipient's native common hepatic artery and the donor's celiac axis or hepatic artery [2,4]. When the patient's hepatic artery is inadequate, interposition grafting securing inflow from the supraceliac or infrarenal aorta has been used [2,4,5]. Donor iliac artery conduits are frequently used for this application. Three studies report successful hepatic artery reconstruction using autogenous splenic artery inflow [4,6,7]. Hepatic artery reconstruction using splenic artery inflow is classically necessitated by proximal dissection within the native hepatic artery requiring access to the native splenic artery [8]. The proximal native splenic artery stump is then anastomosed in an end-to-end fashion to the donor's hepatic artery [8]. The donor celiac axis is sometimes procured and used to form a lengthening vascular conduit [3,4]. Splenic artery adequacy is assessed pre-operatively and intra-operatively. Preoperative computed tomography (CT) allows the determination of splenic artery patency and size. The minimum acceptable diameter of a proposed splenic artery conduit is 4 mm. Intraoperative assessment includes visual and palpable confirmation of patency and Doppler blood flow of greater than 250 ml/minute. We report the Henry Ford Hospital's experience, from January 1996 through January 2007, with seven patients requiring hepatic artery reconstruction during OLT using native recipient splenic artery inflow.

## Case presentation

### Case 1

The first case was a 53-year-old man with cirrhosis of the liver secondary to hepatitis C. During OLT his native hepatic artery was diminutive with minimal flow. The splenic artery was notably tortuous and dilated. Hepatic artery reconstruction was elected using the recipient's native splenic artery. The proximal splenic artery was dissected until a sidebiting vascular clamp was easily accommodated. The donor celiac trunk was then divided and anastomosed to the recipient's splenic artery in an end-to-side fashion. Hepatic arterial inflow measured 350 ml/minute. Approximately 4 years later the patient's liver function tests were normal and ultrasound assessment demonstrated normal arterial flow through the splenic artery reconstruction.

### Case 2

The second case was a 37-year-old man who received an OLT as a consequence of cholestatic hepatic disease. Postoperative fibrosis from previous biliary and pancreatic surgery complicated the hilar dissection. The patient's native hepatic artery was diminutive with intermittent spasm causing poor flow. Palpation of the patient's splenic artery demonstrated an excellent pulse. The splenic artery was then dissected to allow placement of a side-biting vascular clamp. The donor celiac trunk was then anastomosed to the recipient's splenic artery in an end-to-side manner. The resulting arterial flow to the liver measured 410 ml/minute. Dampened waveforms discovered in the postoperative period prompted an arteriogram. Stenosis was found at the origin of the recipient's celiac trunk and not at the splenic artery to donor celiac trunk anastomosis. Balloon dilation and stenting corrected the stenosis. Ultrasound waveforms and liver function studies improved. Graft function was excellent at 19 months' follow-up.

### Case 3

The third case was a 28-year-old woman who received an OLT for fulminant hepatic failure. HAT resulted in bile duct necrosis and infected bilomas. She underwent retransplantation 6 months later. The patient's native splenic artery provided an excellent pulse and caliber. Using a side-biting clamp, the donor celiac trunk was anastomosed to the patient's splenic artery in an end-to-side manner. Inflow then measured 365 ml/minute. The patient did well and was discharged to home. She returned 8 months later with elevated liver enzymes. Angiography demonstrated long segment stenosis of the transplanted hepatic artery secondary to intimal hyperplasia. This stenotic area lay distal to the donor celiac trunk to native recipient splenic artery anastomosis. Angioplasty and stenting successfully relieved the stenosis. Routine follow-up discovered depressed Doppler waveforms with normal liver function tests. Subsequent angiography revealed donor HAT with developed collateral flow. Graft function was excellent at 9 months following retransplantation.

### Case 4

The fourth patient was a 55-year-old man who received an OLT for decompensated cirrhosis complicated by hepatorenal syndrome and morbid obesity. The patient also exhibited complex arterial anatomy but the hepatic artery was judged adequate. The donor celiac trunk was anastomosed to the native recipient hepatic artery. Liver enzymes remained elevated postoperatively. Morbid obesity then compromised ultrasound reliability. Angiography revealed HAT. Open hepatic artery thrombectomy was performed. The splenic artery was dissected for inflow and anastomosed to an autogenous saphenous vein graft fashioned in an end-to-side manner. The saphenous vein conduit was then anastomosed to the donor celiac trunk. Excellent flow and pulse were noted in the hepatic artery as a result. Several intrahepatic strictures developed and necessitated retransplantation 2 months later. The saphenous vein allograft was used for arterial inflow for the retransplantation and the patient recovered well without complications. At follow-up 12 months after retransplant, the patient had developed long segment stenosis in the celiac artery including a segment of the saphenous vein conduit. Angioplasty and stenting successfully treated the stenosis.

### Case 5

The fifth case was a 55-year-old man who developed hepatocellular carcinoma from hepatitis C. At the time of transplantation he was noted to have an intimal dissection in the native hepatic artery that extended close to the origin of the splenic artery. The native splenic artery had an excellent pulse. Given our past experience with postanastomotic stenosis with end-to-side hepatic artery-splenic anastomosis, we elected to proceed with an end-to-end anastomosis. The splenic artery was controlled proximally and divided. The distal stump was ligated twice. The proximal stump was anastomosed to the donor celiac trunk without a patch. Excellent flow was recorded above 400 ml/minute. The patient did well postoperatively and continues to do well 21 months after OLT.

### Case 6

The sixth patient was a 65-year-old woman with primary biliary cirrhosis. At the time of OLT she developed an intimal dissection as described in case 5. She had a very large and well-developed intact splenic artery. The splenic artery was utilized for inflow using the end-to-end technique described above. Recorded hepatic artery inflow was 350 to 400 ml/minute. Postoperative outcome was uneventful and the patient continues to do well with normal hepatic artery flow 19 months after OLT.

### Case 7

The final case was a 59-year-old man who developed cirrhosis as a result of hepatitis C. He had massive ascites with recurrent spontaneous bacterial peritonitis. At the time of OLT, the patient had a very dense hilum with significant sclerosis and a barely palpable hepatic artery pulse. Once isolated, the hepatic artery was diminutive and inadequate for inflow. Preoperative CT scan of the abdomen showed a large and tortuous splenic artery. The native splenic artery was then used for inflow using an end-to-end anastomosis to the donor celiac trunk without a patch. Flow measured 500 to 550 ml/minute. The patient did well and continued to have excellent hepatic artery waveforms 14 months after OLT.

## Discussion

Hepatic artery reconstruction traditionally relies on interposition grafting between the donor hepatic artery and either the recipient's infrarenal or supraceliac aorta. Dissection of the supraceliac and infrarenal aorta can be difficult, especially in the settings of obesity, the presence of collaterals, prior surgery or intra-abdominal adhesions. Infrarenal interposition grafting also carries a higher risk of thrombosis. Both techniques also involve using a donor iliac artery as a conduit, which may not be suitable due to donor issues or prolonged preservation due to an extended interval between OLT and hepatic artery revision. The latter situation is complicated by the degeneration of the arterial conduit, which becomes more significant as time progresses, especially past 3 to 5 days [9].

**Figure 1:**
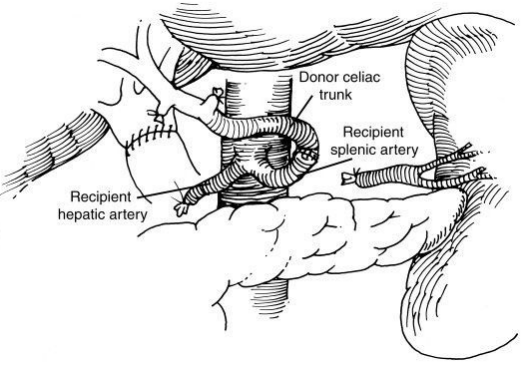
Diagrammatic representation of end-to-end anastomosis between splenic artery and donor celiac trunk.

**Figure 2:**
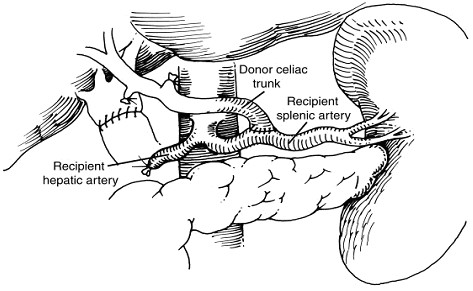
**Diagrammatic representation of end-to-side anastomosis between the recipient splenic artery and donor celiac trunk**.

Figueras et al. described hepatic arterial inflow reconstruction using recipient splenic artery and donor hepatic artery in 1995 [7]. Most cases of splenic artery inflow reconstruction used end-to-end anastomosis (Fig. 1) while a few cases of end-to-side anastomosis (Fig. 2) have been reported [3,4,7]. Splenic infarction and pancreatitis are rare complications of splenic artery ligation not observed in this patient series and possibly obviated by end-to-side anastomosis. Preservation of the short gastric vessels demonstrated significant value, with splenic inflow from the short gastric vessels noted on postoperative arteriograms. Good graft function in this study confirmed the viability of splenic artery inflow reconstruction when the native hepatic artery is inadequate. Ease of access and dissection offered further technical advantages of using the splenic artery instead of the aorta as an inflow source for conduit reconstruction.

**Figure 3:**
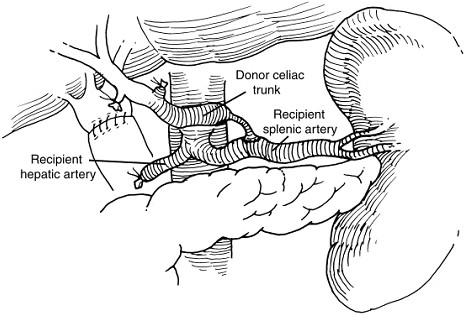
Diagrammatic representation of end-to-side anastomosis between the recipient splenic artery and donor celiac trunk with postanastomotic stricture.

Some of our patients developed postanastomotic donor arterial intimal hyperplasia. The cause of this intimal hyperplasia remains speculative but the possibility of 'splenic steal' or turbulence was suggested. Two cases of postanastomotic arterial stenosis (Fig. 3) resulting from intimal hyperplasia were successfully treated with endovascular angioplasty and stenting. Our institutional experience suggests that the choice of location of arterial anastomosis for inflow may have an impact on the technical outcome and patency of the hepatic artery [10]. Consideration of additional factors that may influence patency and longevity of the established arterial inflow, beyond anastomotic technique, must be considered when performing nonanatomic reconstructions. The creation of new flow diversions, as in end-to-side anastomosis, and new sources of flow with different pulsatility and flow dynamics may also have an impact. Due to the small number of patients, systematic evaluation of all factors contributing to long-term patency would be difficult.

The use of a native saphenous vein for arterial revascularization of hepatic arterial pseudoaneurysms has been established [11,12]. An autogenous saphenous vein was successfully used in one patient in this report to construct the native splenic artery to donor celiac trunk conduit.

## Conclusion

In this case series, we have shared our experience with splenic artery inflow during OLT in seven patients. The initial assumption was that an end-to-side anastomosis to the splenic artery would preserve the spleen and ensure a natural lie with good flow to the hepatic artery. However, although the numbers involved in this series are small, this technique was associated with a relatively high rate of postanastomotic intimal hyperplasia. Adopting the end-to-end technique proved very effective for hepatic inflow while demonstrating splenic and pancreatic safety. Dissection and ligation of the splenic artery did not result in splenic infarction or pancreatitis. The avoidance of surgical dissection and clamping of the aorta was an additional benefit of using the splenic artery. Assessment of the adequacy of the hepatic and splenic arteries with preoperative CT has become our policy. In the event of an inadequate native hepatic artery, the native splenic artery has become our preferred secondary alternative. Aortic conduits have been reserved for situations in which splenic arterial inflow is inadequate. Splenic artery inflow reconstruction is a valuable and effective technique that should be added to the surgeon's armamentarium for performing OLT.

## Abbreviations

CT: Computed tomography; HAT: Hepatic artery thrombosis; OLT: Orthotopic liver transplant.

## Competing interests

The authors declare that they have no competing interests.

## Authors' contributions

WBV directly assembled patient information, directed in-depth analysis of the known literature and drafted the manuscript. MSA served as the most senior staff reviewer and primary investigator, and initially conceived of the possibility of alternative forms of vascular reconstruction. AY served as a senior staff reviewer and primary investigator. DYK served as a junior staff reviewer and primary investigator. MSA, AY and DYK scheduled and immediately directed surgical interventions and supervised the patients' recovery and follow-up. All authors read and approved the final manuscript.

## Consent

Written informed consent was obtained from the patients for publication of this case series. A copy of the written consent is available for review by the Editor-in-Chief of this journal.
